# Resveratrol Attenuates CoCl_2_-Induced Cochlear Hair Cell Damage through Upregulation of Sirtuin1 and NF-κB Deacetylation

**DOI:** 10.1371/journal.pone.0080854

**Published:** 2013-11-21

**Authors:** Ping Wang, Bo Du, Wanzhong Yin, Xinrui Wang, Wei Zhu

**Affiliations:** 1 Department of Otolaryngology-Head and Neck Surgery, First Hospital of Jilin University, Changchun, China; 2 Key Laboratory of Zoonoses, Ministry of Education, Institute of Zoonoses, Jilin University, Changchun, China; Indian Institute of Toxicology Reserach, India

## Abstract

The goals of this study were to investigate the effects of hypoxia on cochlear hair cell damage, and to explore the role of sirtuin1 in hypoxia-induced hair cell damage. Cochlear organotypic cultures from postnatal day 4 rats were used in this study. Hypoxia was induced by treating cochlear explants with CoCl_2_. Cochlear cultures were treated with CoCl_2_ alone or in combination with the sirtuin1 activator resveratrol and the sirtuin1 inhibitor sirtinol. Hair cell damage was identified by phalloidin staining and imaged using scanning electron microscopy. RT-PCR and Western blot analyses were used to detect the expression of sirtuin1 and acetylated nuclear factor-κB (NF-κB). Low concentrations of CoCl_2_ (100–200 μM) did not cause an obvious change in the number and morphology of hair cells, whereas higher concentrations of CoCl_2_ (300–400 μM) induced swelling of hair cells, accompanied by cell loss. Increased sirtuin1 expression was induced by CoCl_2_ at 100 to 200 μM, but not at 400 μM. NF-κB acetylation was significantly increased in explants treated with 400 μM CoCl_2_. Pretreatment with resveratrol prevented CoCl_2_-induced hair cell loss and acetylation of NF-κB. The protective effect of resveratrol was significantly reduced by sirtinol. CoCl_2_ induces hair cell damage in organotypic cochleae cultures. Resveratrol attenuates CoCl_2_-induced cochlear hair cell damage possibly via activation of sirtuin1, which deacetylates NF-κB.

## Introduction

Neonatal hypoxic ischemic encephalopathy, a common disease in perinatal neonates, can cause various neurological sequelae, including epilepsy, cerebral palsy, visual impairment, and mental retardation. Sensorineural hearing loss is a severe complication that is associated with perinatal hypoxia and ischemia [1,2]. Jiang et al. reported that 16.2% of infants with perinatal hypoxia-ischemia also presented with impaired cochlear hair cell function [3]. Ischemia and hypoxia in the inner ear contribute to sudden deafness, acute acoustic trauma, and presbyacusis [4–6]. 

Cochlear tissues are very sensitive to hypoxia. Complex action potentials and endocochlear potentials have been shown to be reduced after transient asphyxia for 45 s in bilateral adrenalectomized rats [7]. Hearing thresholds were elevated in animal models of hypoxic-ischemic encephalopathy, accompanied by apoptosis in the organ of Corti, spiral ganglion cells, and brainstem neurons [8]. Hair cell loss and neuronal apoptosis have been found in cochlear organotypic cultures in *in vitro* models of hypoxia and ischemia [9–11]. Mitochondrial cristae in cochlear inner hair cells (IHCs) disappeared in rats weaned from mechanical ventilation for 10 min. Huge vacuoles occurred in the IHCs, accompanied by swollen cell bodies after hypoxia for 30 min. Most IHCs exhibited ruptured cell membranes with a leakage of intracellular contents, accompanied by vacuoles in IHCs after hypoxia for 60 min [12].

The mechanism of hypoxia-induced IHC damage remains unclear. Hypoxia may induce hair cell loss by increasing intracellular Ca^2+^ levels due to disruption of ATP-dependent Ca^2+^ regulatory mechanisms [13]. Hypoxia can also induce the expression and secretion of proinflammatory cytokines in explanted cochlear tissues [14], suggesting that inflammation may contribute to hypoxia-induced hair cell loss.

Silence information regulation 1 (Sirt1), a NAD-dependent histone deacetylase, is extensively expressed in the cytoplasm and nucleus. Sirtuin1 plays an important role in cellular transcription and metabolism, and it has anti-apoptotic and anti-aging properties. Sirtuin1 overexpression was found to protect neurons from oxidative damage and inhibit neurodegeneration by promoting the transcriptional activity of PGC-1α and increasing mitochondrial density [15]. Sirtuin1 was also found to inhibit cell apoptosis by regulating many non-histone substrates, such as FOXO, tumor necrosis factor (TNF)-α, nuclear factor-κB (NF-κB), and p53 [16,17]. Sirtuin1 binds to the RelA/p65 subunit of NF-κB and inhibits acetylation of RelA/p65 at lysine 310, thereby decreasing NF-kB activation [18,19]. Thus, regulation of p65 acetylation by sirtuin1 may be a potential target for the treatment of inﬂammatory injury. 

The role of sirtuin1 and acetylation of NF-κB p65 in hypoxia-induced cochlea injury remain unclear. In this study, we investigated hypoxia-induced hair cell damage in cochlear organotypic cultures from postnatal day 4 (P4) rats, using a CoCl_2_-induced hypoxic model. The aims of this study were to investigate the effects of hypoxia on the cochlear hair cell damage, and to explore the role of sirtuin1 in hypoxia-induced hair cell damage.

## Materials and Methods

### Cochlear organotypic culture

This study was performed in accordance with the institutional recommendations for animal care of Jilin University. The care and use of the animals in this study was approved by the Jilin University Animal Care and Use Committee. All Wistar rats were purchased from the experimental animal center of Basic Medical College of Jilin University. The Institutional Animal Care and Use Committee of Jilin University approved this study.

Wistar rats of postnatal day 4 (P4) were prepared by inhalation anesthesia with 4% halothane. The skull was opened, and the temporal bones were harvested under sterile conditions. Cochleae containing the organs of Corti were removed by dissection of the spiral ligament and internal nerve fibers. The basilar membrane was placed on poly-lysine–coated culture dishes, with care taken to maintain the natural curvature of the cochlea. 

Cochleae were cultured in DMEM/F12 culture medium (Invitrogen, USA) with N2 supplements containing 10% fetal bovine serum (FBS, Invitrogen, USA). After 24 h in culture, the cochleae were treated with 100, 200, 300, and 400 μM CoCl_2_ (dissolved in DMEM/F12 culture medium) for 24 or 48 h. DMEM/F12 culture medium alone was used as a vehicle control. To investigate the effects of resveratrol on CoCl_2_-induced activation of sirtuin1, cochleae were pretreated with 50 μM resveratrol for 1 h and cultured in DMEM/F12 medium containing 400 μM CoCl_2_ for 24 h.

### Phalloidin staining

To visualize the cellular structure of the cochleae, F-actin was stained with TRITC-labeled phalloidin. Briefly, after culture for 24 h, cochleae were fixed in 10% formaldehyde in PBS overnight at room temperature. Cochleae were then washed with 0.1 M PBS three times, and treated with 0.25% Triton X-100 for 10 min. They were stained with TRITC-labeled phalloidin (1: 200, Sigma-Aldrich, USA) for 30 min. After three washes with PBS, the specimens were counterstained with Hoechst 33342, and viewed using confocal microscopy. 

Hair cells were counted in a 160-μm-long section in three different zones located at the apical, middle, and basal turns of each organ of Corti. Three sections of each zone were counted, and the average of the three zones was determined. Hair cell loss was calculated as the percentage of missing hair cells to the total hair cells in each zone.

### Scanning electron microscopy

The basilar membrane of each cochlea was placed on a coverslip and cultured in DMEM/F12 culture medium, alone or with 400 μM CoCl_2_, in a 35-mm culture dish. Samples for scanning electron microscopy (SEM) were prepared as previously described with some modifications [20]. Briefly, after culture, the basilar membrane of cochleae were washed with PBS for 5 min and fixed with 2.5% glutaraldehyde for 24 h. After three 10-min washes with 0.13 M PBS, they were postfixed in 1% OsO4 for 2 h, dehydrated in a graded series of ethanol (50%, 70%, 80%, 90%, and 100% for 10 min each), and then incubated in tert-butoxide for 10 min. Samples were critical-point dried with CO_2_, coated with gold (Au) using ion sputter coater, and viewed and imaged with a scanning electron microscope. 

### Real-time quantitative RT-PCR

Total RNA was isolated from cochlear organotypic cultures by using Trizol reagent (Invitrogen, USA) according to manufacturer’s protocol. RNA was reverse-transcribed into complementary DNA (cDNA) using the Quantscript reverse transcription kit (Tiangen, Beijing), according to the manufacturer’s instructions. RT-PCR was performed in a final volume of 20 μl mixture containing 5 μl of cDNA, 5 μl of each primer, and 10 μl of Mater SYBRGreen I mix (Roche, USA). Primers were selected as previously described [8]. Primers were 5’-TGTTTCCTGT GGGATACCTG A-3’ (forward) and 5’- TGAAGAATGG TCTTGGGTCT TT-3’ (reverse) for sirtuin1 (size, 137 bp). β-actin was used as an internal control. The reaction conditions were as follows: 95 °C for 10 min; 94 °C for 30 s, 60 °C for 30 s, and 72 °C for 45 s with 35 cycles; and 72 °C for 7 min. Melting curve analyses were performed to verify the amplification specificity. The gene expression ΔCt values of sirtuini1 mRNAs from each sample were calculated by normalizing with internal control actin. The relative expression of sirtuin1 was calculated using 2-ΔΔCT method [20]. 

### Western blot

After culture for 24 h, the basilar membrane of the cochlea was homogenized on ice in lysis buffer. Lysates were centrifuged at 12,000 rpm at 4 °C for 15 min. Protein concentrations were determined using the BCA method. Proteins (30 μg) were resolved by SDS–PAGE and transferred onto polyvinylidene fluoride membranes by electroblotting. Membranes were incubated with 5% fat-free milk for 2 h, followed by primary antibodies against sirtuin1 (dilution 1:500, Santa Cruz, USA) and acetylated NF-κB (dilution 1:1000, Cell Signaling, USA) at 4 °C, overnight. β-actin was used as a loading control. Membranes were incubated with horseradish peroxidase-linked goat anti-rabbit secondary antibodies (dilution 1:2000) at room temperature, for 2 h. Bands were visualized using a chemiluminescence detection system, and analyzed using Quantity One software (version 4.52). 

### Statistical analyses

Analyses were performed using SPSS (version 17.0). All values are presented as mean and standard deviation (SD). One-way or two-way analysis of variance (ANOVA) was used to compare differences in the percentage of hair cell loss, or the expression of sirtuin1 and NF-κB among two or more groups, respectively, followed by Turkey’s post hoc tests. Probability values less than 0.05 were considered statistically significant. 

## Results

### CoCl_2_ induces hair cell damage in the organotypic culture of the cochlea

**Figure 1:**
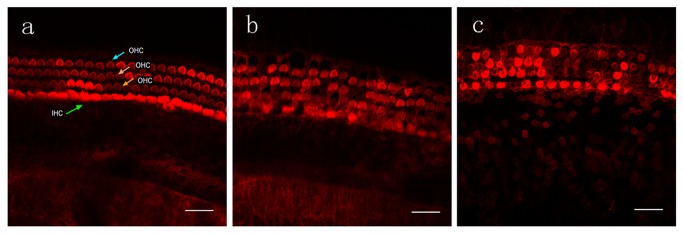
Hair cell death induced by CoCl_2_ exposure. Cochlear explant cultures from P4 rats were treated with different concentrations of CoCl_2_ for 24 h. Explants were stained with TRITC-labeled phalloidin and observed using confocal microscopy. A. Representative micrograph of control explants showing an orderly row of IHCs and three rows of OHCs with stereocilia bundles. B. Representative micrograph of explants exposed to 300 μM CoCl_2_ showing loss of IHCs and OHCs. C. Representative micrograph of explants exposed to 400 μM CoCl_2_ showing loss of a large number of hair cells with widened intercellular spaces. Bar, 20 μm. IHC, inner hair cell; OHC, outer hair cell.

Organ of Corti explants from P4 rat cochlea were treated with different concentrations of CoCl_2_ for 24 h, stained with TRITC-labeled phalloidin, and observed using a confocal microscope (Figure 1). Control explants exhibited a normal pattern of three rows of outer hair cells (OHCs) and a single row of IHCs (Figure 1). Hair cells were arranged in an orderly manner, and the structure of the sterocilla bundles was clearly observed. 

**Figure 2:**
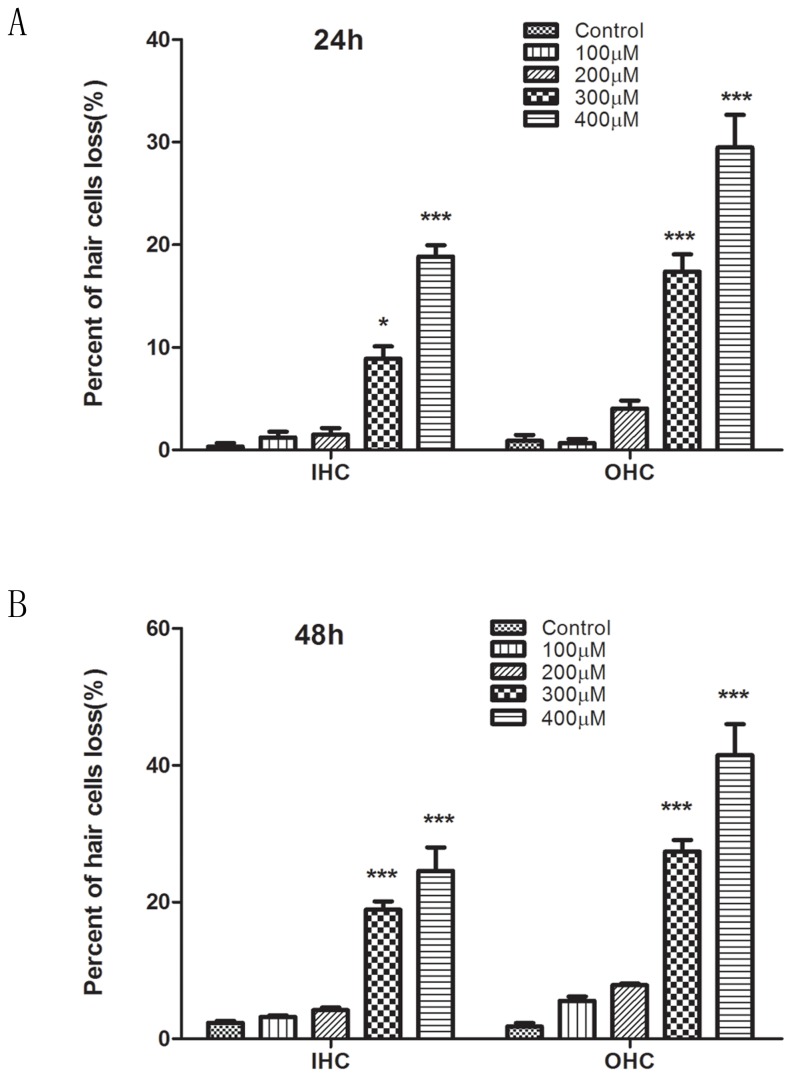
Quantitative analysis of hair cell loss in explants treated with CoCl_2_. (A) CoCl_2_ treatment for 24h. (B) CoCl_2_ treatment for 48h. The percentage of IHC and OHC loss was calculated in the apical, middle and basal turns, and the average of the three zones were presented. N = 5 for each group. * *P* < 0.05, ^**^ *P* < 0.01 versus control, 100 μM CoCl_2_ or 200 μM CoCl_2_.

**Figure 3:**
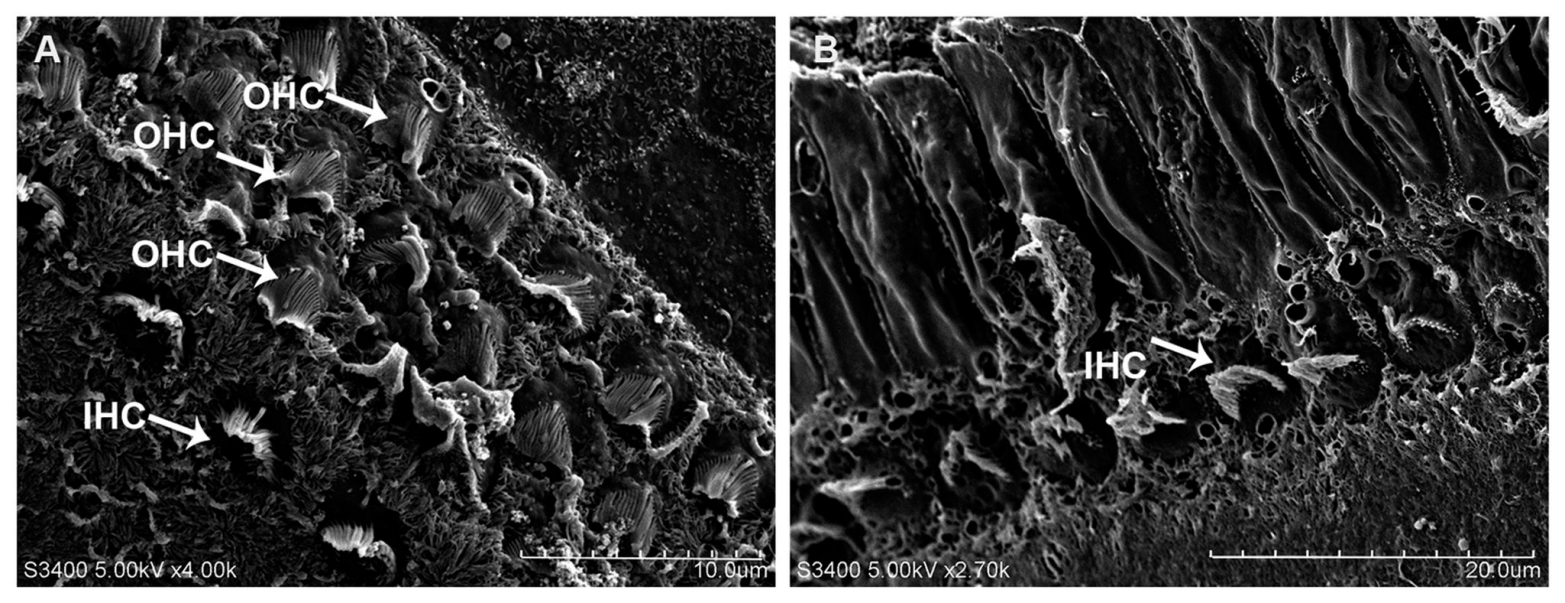
Scanning electron microscopy results showing hair cells in the basal zone of control explants. (A) and explants treated with 400 μM CoCl_2_ for 24 h (B). After CoCl_2_ treatment, most OHCs were lost, while the structure of most IHCs remained. The basilar membrane was fused to form a cord-like structure.

Low concentrations of CoCl_2_ (100–200 μM) did not cause obvious changes in the number or morphology of hair cells, whereas 300 μM CoCl_2_ induced swelling of OHCs accompanied by cell loss. After treatment with 400 μM CoCl_2_, a large number of hair cells were lost, and the orderly structure was disrupted. CoCl_2_ dose-dependently induced cell loss of both IHCs and OHCs (Figure 2). Exposure to 400 μM CoCo_2_ for 48 h significantly increased the percentage losses of IHCs (24.54%) and OHCs (41.48%) compared to exposure for 24 h (IHC loss, 18.84%; OHC loss, 29.48%). SEM results confirmed that CoCl_2_ induced more OHC loss than IHC loss (Figure 3). After 400 μM CoCl_2_ exposure for 24 h, most OHCs were lost, and the basilar membrane was fused, but most IHCs survived (Figure 3).

### CoCl_2_ induces upregulation of sirtuin1

**Figure 4:**
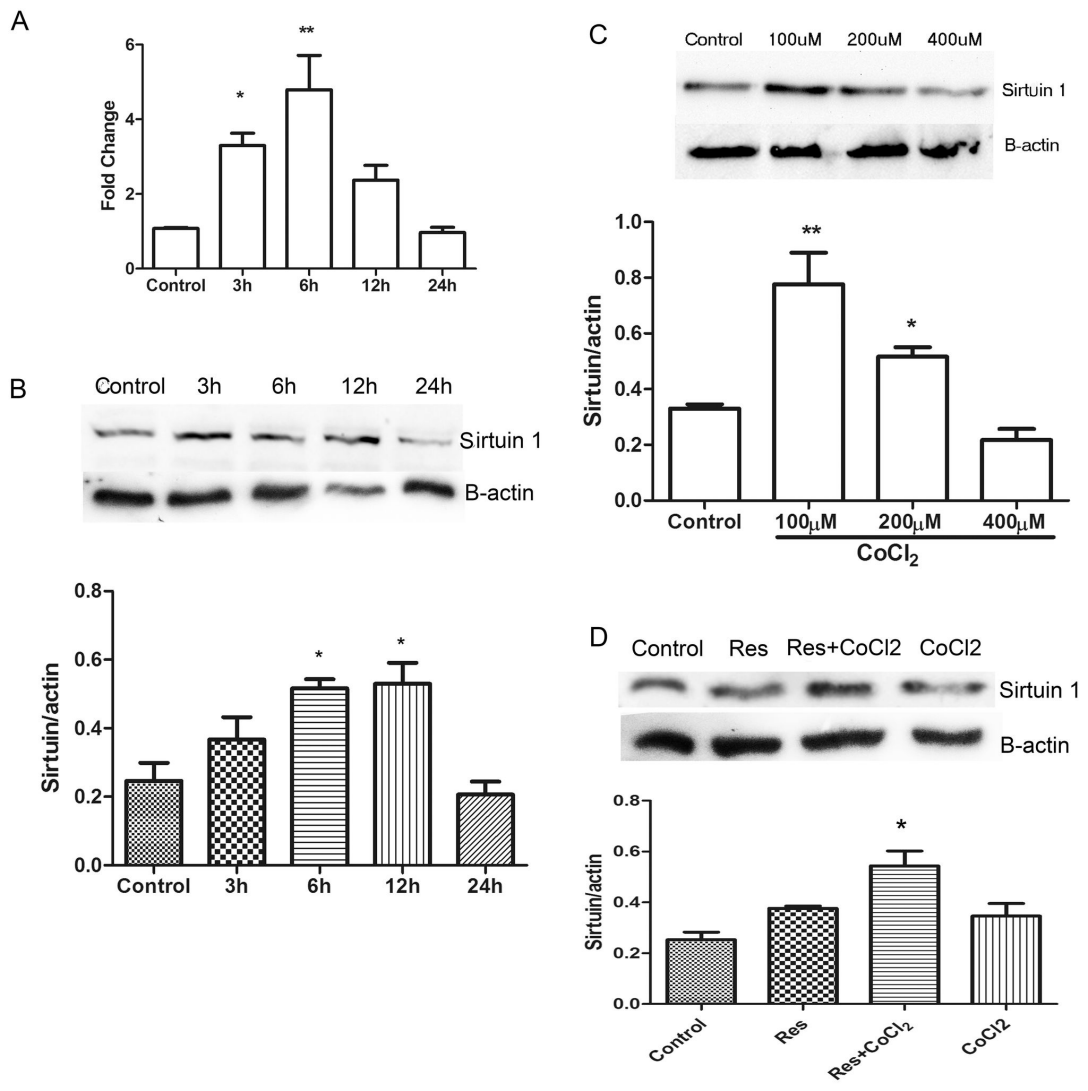
CoCl_2_ upregulates the mRNA and protein expressions of sirtuin1. (A) Real-time quantitative RT-PCR showing relative mRNA expression of sirtuin1 in cochlear explants treated with 400 μM CoCl_2_ for 3, 6, 12, and 24 h. Expression of β-actin was determined as an internal control. * *P* < 0.05 versus control, ** *P* < 0.01 versus control. (B) Western blot showing the protein expression of sirtuin1 in cochlear explants treated with 400 μM CoCl_2_ for 3, 6, 12, and 24 h. * *P* < 0.05 versus control. (C) Representative Western blot showing the protein expression of sirtuin1 in cochlear explants treated with different concentrations of CoCl_2_ for 24 h. Quantitative Western blot analysis of the protein expression of sirtuin1. β-actin was used as a loading control. * *P* < 0.05, ^**^ *P* < 0.01 versus control. (D) Representative Western blot showing the protein expression of sirtuin1 in cochlear explants pretreated with 50 μM Resveratrol for 1 h, followed by exposure to 400 μM CoCl2 for 24 h. * *P* < 0.05 versus CoCl_2_ treatment.

We investigated the effects of 400 μM CoCl_2_ treatment on the expression of sirtuin1 in organotypic cultured cochlea, using real-time RT-PCR and Western blot analyses. Compared with the control, the mRNA expression of sirtuin1 was significantly increased in explants treated with CoCl_2_ for 3 h. Sirtuin1 mRNA levels reached a peak in explants treated with CoCl_2_ for 6 h, and declined to a level lower than the control in explants treated with CoCl_2_ for 24 h (Figure 4A). Western blot analysis showed that the protein expression of sirtuin1 was significantly increased in explants treated with CoCl_2_ for 3 h, reached a peak in explants treated with CoCl_2_ for 12 h, and declined to a level lower that the control in explants treated with CoCl_2_ for 24 h (Figure 4B). We tested the effects of different concentrations of CoCl_2_ on the protein expression of sirtuin1. After exposure to CoCl_2_ for 24 h, low concentrations of CoCl_2_ (100 and 200 μM) significantly increased the expression of sirtuin1 compared with the control. However, the highest concentration of CoCl_2_ (400 μM) did not upregulate the expression of sirtuin1 (Figure 4C). In addition, resveratrol (50 μM) pretreatment significantly increased the expression of sirtuin1 in explants treated with 400 μM CoCl_2_ for 24 h (Figure 4D).

### Resveratrol attenuates CoCl_2_-induced hair cell damage

**Figure 5:**
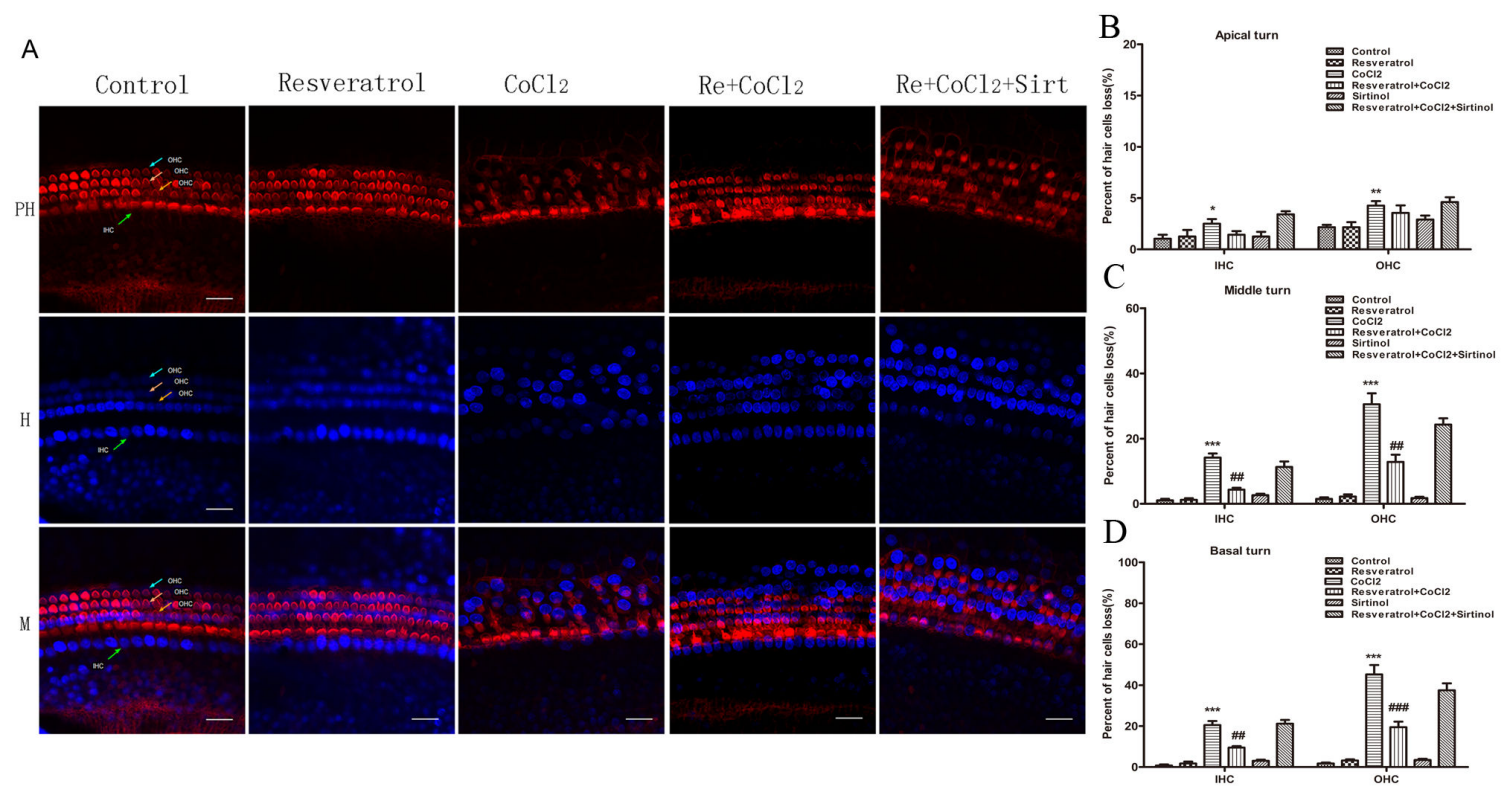
Resveratrol attenuates CoCl_2_-induced hair cell damage. (A) Representative micrographs of cochlear explants stained with TRITC-labeled phalloidin (upper), Hoechest 33342 (middle), and their merged images (lower). Control explants cultured in normal culture medium for 24 h (*n* = 6); resveratrol, explants pretreated with 50 μM resveratrol for 1 h (*n* = 6); CoCl_2_, explants treated with 400 μM CoCl_2_ for 24 h (*n* = 6). Re + CoCl_2_, explants pretreated with 50 μM resveratrol for 1 h followed by exposure to 400 μM CoCl_2_ for 24 h (*n* = 6). Re + CoCl_2_ + Sirt, explants pretreated with 50 μM resveratrol for 1 h followed by exposure to 400 μM CoCl_2_ and 20 μM sirtinol. Bar, 20 μm. B-D. Percentage of IHC and OHC loss in the apical (B), middle (C), and basal (D) turns. Hair cells in three 160 μm-long sections at each zone were calculated. The average was used to calculate the percentage of IHC and OHC loss. * *P* < 0.05, ^**^ *P* < 0.01, ^***^ *P* < 0.001 versus control; ^##^ *P* < 0.01, ^###^ *P* < 0.001 versus CoCl_2_.

We investigated the effect of the sirtuin1 activator resveratrol and the sirtuin1 inhibitor sirtinol on CoCl_2_-induced hair cell damage. In cochlear explants treated with 400 μM CoCl_2_ for 24 h, hair cells exhibited a base-to-apex gradient damage, with the greatest degree of damage in the basal turn (Figure 5). The percentage losses of IHCs and OHCs were both <5% in the apical turn, 14.17% and 30.55% in the middle turn, and 20.53% and 45.19% in the basal turn, respectively. 

Pretreatment with resveratrol significantly decreased the CoCl_2_-induced IHC and OHC losses in the middle and basal turns. The protective effect of resveratrol was significantly reduced by sirtuin1 inhibitor sirtinol. These results suggest that the upregulation of sirtuin1 plays a protective role in CoCl_2_-induced hair cell loss.

### Resveratrol inhibits CoCl_2_-induced NF-κB acetylation

**Figure 6:**
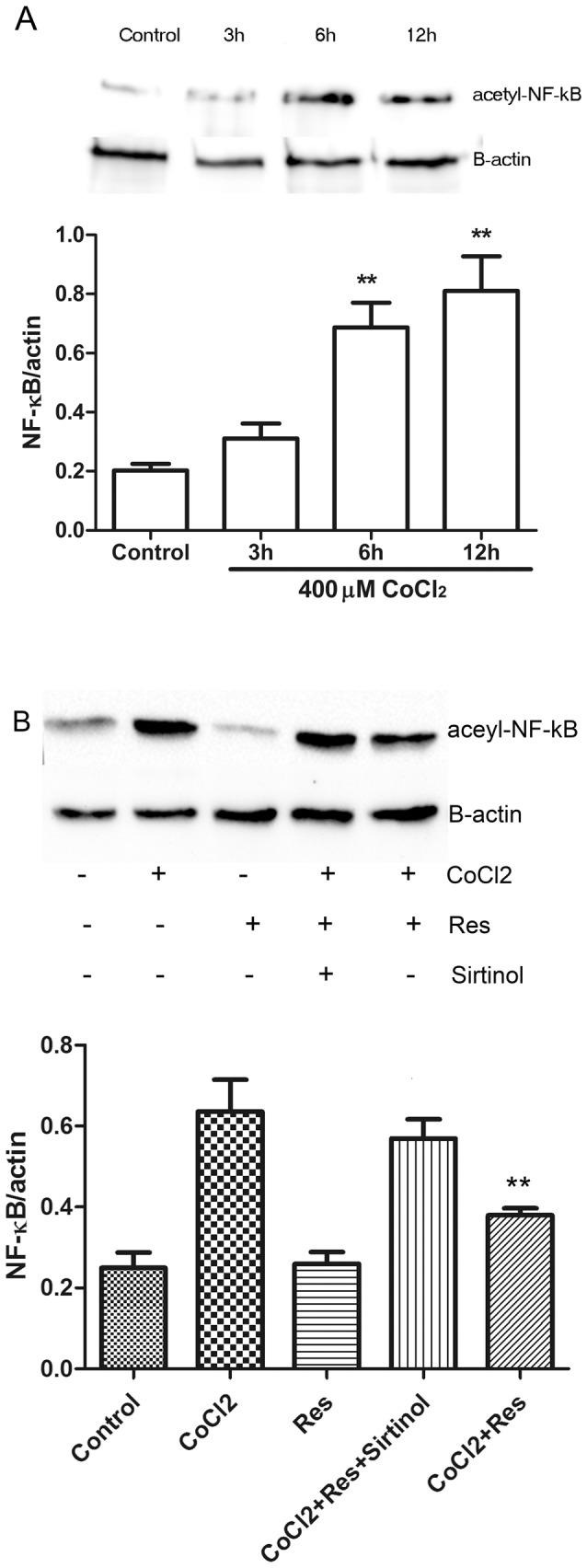
Expression of acetylated NF-κB in explants treated with CoCl_2_. (A) Representative Western blot showing the expression of acetylated NF-κB in cochlear explants treated with 400 μM CoCl_2_ for 3, 6, and 12 h. Quantitative Western blot analysis of the protein expression of acetylated NF-κB in B. β-actin was used as a loading control. **P < 0.01 versus control. (B) Representative Western blot showing the expression of acetylated NF-κB in control explants, explants treated with 400 μM CoCl_2_ for 24 h, explants pretreated with 50 μM resveratrol for 1 h, explants pretreated with 50 μM resveratrol for 1 h, followed by exposure to 400 μM CoCl_2_ for 24 h, and explants pretreated with 50 μM resveratrol for 1 h, followed by exposure to 400 μM CoCl_2_ plus 20 μM sirtinol for 24 h. Quantitative Western blot analysis of the protein expression of acetylated NF-κB. β-actin was used as a loading control. **P < 0.01 versus CoCl_2_.

Sirtuin1 can bind to and deacetylate NF-κB, leading to inhibition of NF-κB activity [19]. To explore the signaling pathway of sirtuin1 in reducing CoCl_2_-induced hair cell damage, we investigated the effect of resveratrol on NF-κB acetylation (Figure 6A). Compared with control explants, NF-κB acetylation was significantly increased in explants treated with 400 μM CoCl_2_ for 6 and 12 h. Pretreatment of resveratrol prevented CoCl_2_-induced acetylation of NF-κB. The effect of resveratrol on CoCl_2_-induced acetylation of NF-κB was significantly reduced by the sirtuin1 inhibitor sirtinol (Figure 6B).

## Discussion

Because *in vivo* animal models of hypoxia can induce hypoxia in many brain areas that may contribute to hypoxia-induced cochlear damage, investigators have adopted *in vitro* models of hypoxia using cochlear organotypic cultures [14,21]. CoCl_2_ is commonly used to induce a hypoxic environment by replacing Fe^2+^ in hemoglobin to form deoxygenated hemoglobin [22–24]. Co^2+^ has been shown to inhibit HIF-lα aryl hydrocarbon-hydroxylase activity and reduce HIF-lα degradation [25]. CoCl_2_ has been used for hypoxic preconditioning in many cell models of hypoxia, but very high concentrations of CoCl_2_ have been found to induce cellular damage by increasing intracellular reactive oxygen species (ROS), decreasing mitochondrial membrane potentials, and inducing cell apoptosis in many cells, especially neurons [26–28]. 

In the present study, we used CoCl_2_ to induce hypoxia in organotypic cultured cochlear explants. Higher concentrations of CoCl_2_ (300 and 400 μM) induced IHC and OHC loss, similar to findings made in an animal model of hypoxia [12]. Furthermore, CoCl_2_ induced more OHC than IHC loss, consistent with previous reports showing that mitochondrial swelling and intracellular vacuoles occurred earlier in OHCs than IHCs in an animal model of hypoxia [29]. However, Amarjargal et al. demonstrated that IHCs were more susceptible to hypoxia than OHCs in an *in vitro* model of oxygen-glucose deprivation-induced hypoxia [9]. This difference is likely due to the different hypoxic models used in the different studies. CoCl_2_-induced chemical hypoxia is thought to be associated with oxidative stress [30,31], which leads to production of a large amount of ROS, subsequent cell apoptosis, and hair cell death. The higher sensitivity of OHCs to hypoxia may be caused by a lower concentration of glutathione in OHCs compared to IHCs, thereby resulting in a weaker capacity to clear ROS [32].We also examined the expression of sirtuin1 in cochlear explants treated with CoCl_2_ over various durations. The mRNA expression of sirtuin1 was significantly increased in explants treated with CoCl_2_ for 3 h, peaked in explants treated for 6 h, and declined in explants treated for 12 h to a level higher than that in controls. Sirtuin1 protein expression patterns showed a similar time course as the mRNA expression, with a delayed peak expression. The protein expression of sirtuin1 increased after treatment with lower concentrations of CoCl_2_ (< 200 μM), but decreased after treatment with the highest concentration of CoCl_2_ used (400 μM). These results suggest that hypoxic preconditioning induces the upregulation of sirtuin1 in the cochlea. In addition, we found that 400 μM CoCl_2_ significantly induced IHC and OHC loss, accompanied by a significant down regulation of sirtuin1. This suggested that sirtuin1 down regulation is the main mechanism underlying CoCl_2_-induced hair cell loss. Furthermore, we found that 200 μM CoCl_2_ significantly increased sirtuin1 expression, and induced more (but not significantly more) hair cell loss compared with controls, suggesting that CoCl2 may induce hair cell loss via other signaling pathways that do not involve sirtuin1. For example, it has been reported that CoCl_2_ induces PC12 cell apoptosis via p53 stability and regulating UCN5B [33]. Further studies are needed to confirm the presence of these signaling pathways in hair cells.

The sirtuin1 activator resveratrol improved survival of IHCs and OHCs. This effect was blocked by the sirtuin1 inhibitor sirtinol. These findings suggest that the activation of sirtuin1 protects cochlear hair cells from hypoxic injury induced by CoCl_2_. Although it has been reported that overexpression of sirtuin1 can result in increased apoptosis and hypertrophy in the heart [34], resveratrol use has also been found to prevent cisplatin-induced ototoxicity [35]. This protective role of resveratrol may be mediated by its ability to decrease the intracellular ROS content in IHCs and OHCs [36]. However, it remains to be determined whether resveratrol improves hair cell survival via its anti-oxidative property.

NF-κB is a target of sirtuin1, and NF-κB acetylation is critical for its activity, especially during inflammation [37]. NF-κB activation promotes inflammatory cytokine production during inflammation, and hypoxia can induce inflammation [38]. Sirtuin1 physically interacts with the RelA/p65 subunit of NF-κB and inhibits transcription by deacetylation of RelA/p65 at lysine 310 [39]. Acetylation of RelA/p65 at lysine 310 is required for the full transcriptional activity of NF-κB during inflammation [40]. In the present study, we found that NF-κB acetylation was significantly increased in explants treated with 400 μM CoCl_2_ for 6 and 12 h, and pretreatment with resveratrol prevented CoCl_2_-induced acetylation of NF-κB. This finding suggests that sirtuin1 activation deacetylates NF-κB and reduces the hypoxia-induced increase in NF-κB activity, thereby protecting cochlear hair cells from hypoxic injury.

In summary, we found that CoCl_2_-induced hypoxia resulted in cochlear hair cell loss in cochlear organotypic cultures from P4 rats. CoCl_2_-induced hair cell loss was prevented by pretreatment of sirtuin1 activator resveratrol, possibly via NF-κB deacetylation. Sirtuin1 activation may represent a new therapeutic target for the prevention or treatment of hypoxia-induced cochlear hair cell injury.
